# Cytokine secretion from brain macrophages infected with human immunodeficiency virus in vitro and treated with raltegravir

**DOI:** 10.1186/1471-2334-14-386

**Published:** 2014-07-11

**Authors:** Erick T Tatro, Benchawanna Soontornniyomkij, Scott L Letendre, Cristian L Achim

**Affiliations:** 1 Department of Psychiatry, University of California San Diego, 9500 Gilman Drive, La Jolla, San Diego, CA 92093-0603, USA; 2 Department of Medicine, University of California San Diego, 200 West Arbor Drive, San Diego, CA 92103-8208, USA

**Keywords:** Microglia, Human immunodeficiency virus, Integrase inhibitor, Raltegravir, IL-10, IL-8, TNF-α

## Abstract

**Background:**

Integrase inhibitors are a promising class of antiretroviral drugs to treat chronic human immunodeficiency virus (HIV) infection. During HIV infection, macrophages can extravasate from the blood to the brain, while producing chemotaxic proteins and cytokines, which have detrimental effects on central nervous system cells. The main goal of this study was to understand the effects of raltegravir (RAL) on human brain macrophage production of immune-mediators when infected with HIV, but did not compare with other antiretroviral agents.

**Methods:**

Pro-inflammatory cytokines, IFN-γ, IL-10, IL-12-p70, IL-1, IL-8, TNF-α, and IL-6 were measured simultaneously in tissue culture supernatants from primary brain derived macrophages, microglia. We tested the effects of RAL on markers of astrocytosis and neurite integrity in primary human neuroglial cultures.

**Results:**

RAL administered at 20 nM effectively suppressed HIV infection in microglia over 9 days. Only IL-8, IL-10, and TNF-α were above the detection limit in the majority of samples and RAL significantly suppressed the rate of cytokine production in HIV-infected microglia. During RAL-alone, the rate of IL-8 secretion was higher.

**Conclusions:**

RAL did not affect neurite area but inhibited astrocyte growth in the neuroglial cultures. Exploring the effects of RAL on pro-inflammatory molecule production in brain macrophages may contribute to designing ARV neuroprotective strategies in chronic HIV infection.

## Background

Chronic human immunodeficiency virus (HIV) infection has detrimental effects on central nervous system (CNS) neuronal health. The principal targets of productive HIV infection in the brain are microglia and monocyte derived macrophages [1-4]. In the periphery, HIV infection in CD4 T cells is cytopathic, resulting in fast CD4 T cell depletion [5], whereas in resting memory T cells, tissue monocytes, macrophages, and brain microglia with integrated HIV survive longer and serve as an HIV reservoir that persists indefinitely [6]. In adult humans, parenchymal microglia are infrequently exchanged with peripheral blood monocytes [7]. Perivascular cells in the CNS with macrophage markers have a higher turnover and are more frequently replaced by blood monocytes. Therefore, perivascular brain macrophages may serve as a source of HIV in the brain, and immunohistological and DNA hybridization studies have confirmed the presence of HIV proteins and DNA in these cells [8], which has been shown to be associated with depleted pre- and post- synaptic markers, synaptophysin and microtubule associated protein-2 (MAP2) [9].

HIV effects on the CNS have behavioral consequences. Neurocognitive impairment in HIV infected individuals is associated with elevated viral load and microglial activation [10,11]. There are several prevailing hypotheses for the mechanisms of neural damage in HIV: direct neurotoxic effect from viral proteins and neural damage via intracellular signaling and secondary neural damage from cytokines and chemokines from infected microglia and brain macrophages [12]. Drugs used to treat HIV have variable neurotoxic effects, as examined by MAP2 staining in neuronal cultures, ranging from 17% to 52% reduction in MAP2 area. Of six combination regimens which included protease inhibitor, non-nucleoside reverse transcriptase inhibitor, and reverse transcriptase inhibitor, four caused significant reduction in neuronal MAP2 area [13]. As new antiretroviral drugs become available, it is important to continue assessing neurotoxicity.

Integrase inhibitors are a relatively new class of antiretroviral compounds, one of which is raltegravir (RAL), which has potent and durable antiviral activity similar to that of efavirenz, but which achieved HIV suppression to below detectable limits at a faster rate in a 24 week study [14]. HIV integration into the host genome is a multistep process involving: a) 3′ endonucleolytic cleavage of the 3′ end of DNA in the HIV pre-integration complex to a conserved CA dinucleotide, b) Strand-transfer involving concerted cleavage of host DNA and ligation of viral 3′ DNA to 5′ staggered site on host DNA, and c) DNA repair of gaps and DNA synthesis. RAL functions by inhibiting the strand-transfer step [15], thus preventing the induction of DNA repair machinery which is associated with innate immune activation and cell death [16]. We were therefore interested in determining the CNS efficacy of RAL by assessing: 1) neurotoxicity using in vitro neuronal cultures following methods comparable to a previous study [13], 2) inhibition of HIV infection in cultured microglia, and 3) cytokine production in RAL-treated, HIV infected microglia.

In primary human neuroglial cultures, we found that RAL is not neurotoxic and we present evidence for reduced astrogliosis. In primary human HLA-DR-positive brain macrophages and microglia, we measured secretion of seven cytokines across 9 d of in vitro HIV infection, and found reduced rate of TNF-α, IL-10, and IL-8 production in those treated with 20 nM RAL.

## Methods

### Cell culture

This study was approved by University of California San Diego Human Research Protections Program. Microglia were isolated through differential adhesion procedure from fetal human brain tissue from elective terminated pregnancy between 12 and 16 weeks of gestation [17], acquired from Advanced Bioscience Resources, donors gave written informed consent for research-use of the cells and tissue. Single-cell suspension of central nervous system cells were plated at 10^8^ cells/mL in a 125 cm^2^ flask and after 7 d, microglia removed by agitation and withdrawal of nonadherent cells which were plated in selection media on coverslips coated with poly-L-lysine (Life Technologies P4707, Carlsbad, CA, USA). Microglia media components were purchased from Life Technologies and consisted of DMEM (11965–092), supplemented with glutamax (35050–061), and gentamicin sulfate (15710–064). After 4 d in culture, cells were differentiated with granulocyte/macrophage colony stimulating factor (GM-CSF, Fisher Scientific, 5056909) for 1 d. Thereafter, no media contained GM-CSF. Microglia were inoculated with a seed stock of HIV_Ba-L_ (5,000 pg/mL HIV p24) in fresh media for 4 hr, then media changed in the presence and absence of 20 nM RAL and maintained without media changes for 9 d. The following reagents were obtained through the NIH AIDS Reagent Program, Division of AIDS, NIAID, NIH: HIV-1_Ba-L_ from Dr. Suzanne Gartner, Dr. Mikulas Popovic and Dr. Robert Gallo [18] and RAL (Cat # 11680) from Merck & Company, Inc. All experimental conditions were performed with three independent replicates.

Aliquots of 60 μL supernatant were removed every 48 hr starting 1 d after infection and stored with 2.5 μL 25X Complete protease inhibitor (Roche 04693116001, Indianapolis, IN, USA) and stored at -80°C to measure cytokine production. The 60 μL was replaced with fresh media (no GM-CSF supplemented) to maintain a constant 500 μL per well. To verify HIV infection, a 200 μL aliquot of supernatant was removed and replaced with fresh media for HIV p24 ELISA at 1 d of infection and at the endpoint (9 d) (Advanced Bioscience Laboratories, Inc. 5421, Rockville, MD, USA). After 9 d, cells were fixed in 4% paraformaldehyde (Electron Microscopy Sciences 15710-S, Hatfield, PA, USA) for microscopy.

To assess for cytotoxicity, 25 μL supernatant at endpoint (9 d) was used to measure lactate dehydrogenase (LDH) activity following manufacturer’s instructions of CytoTox 96 Non-radioactive Cytotoxicity Assay (Promega, Madison, WI, USA). Percent-cytotoxicity was calculated for each condition by comparing to LDH activity of supernatant from cells lysed in 0.1% Triton-X 100 for 1 hr.

Neuronal cultures were generated according to a protocol first described by White et al. [19] and modified for our current usage exactly as described by Nguyen et al. [20] from donated fetal human brain tissue from elective terminated pregnancy between 12 and 16 weeks of gestation. Single-cell suspension of central nervous tissue were plated at 10^5^ cells/mL on glass coverslips coated in poly-L-orinithine and laminin in 24 well-plates. Cultures were mixed neuron - glia culture composed of approximately 60% neurons and 40% astrocytes. After 28 d in culture, neurons were exposed to 20 and 100 nM RAL overnight following by fixation and microscopy analysis.

### Microscopy

Cells were fixed in 4% paraformaldehyde for 10 min at 37°C and washed 3 times in Tris buffered saline (PBS). Cells were permeablized and non-specific antigen blocked using 0.2% Triton X-100 and 2% fetal bovine serum for 2 hr, then incubated overnight with primary antibody at 4°C. After washing 3 times in TBS-T (0.2% Tween-20), secondary antibodies were incubated at 1:750 dilution in blocking buffer, then washed 3 times in TBS-T, a final wash in distilled water, then finally mounted on glass slides with Vectashield with DAPI mounting media (Vector Labs H-1500, Burlingham, CA, USA). Images were captured by laser scanning confocal microscopy at 40X magnification for quantification, images were captured for 10 random fields per coverslip. The imaging conditions were maintained exactly the same across the different experimental conditions (separately for measuring β-III tubulin, GFAP, or NF-κ-B). The following primary antibodies were used, diluted in blocking buffer: mouse anti β-III tubulin (1:400) (R&D Systems MAB1195, Minneapolis, MN, USA), rabbit anti glial fibrillary acidic protein (GFAP) (1:5,000) (Dako, Z0334, Glostrup, Denmark), rabbit anti phospho-NF-kB (1:200) (Cell Signaling Technologies 3033, Billerica, MA, USA), and mouse anti HLA-DR (1:200) (Abcam ab17101, Cambridge, MA, USA). Secondary antibodies used were the following: Alexafluor-568 conjugated sheep anti mouse IgG (Life Technologies 11031) and Alexafluor-488 conjugated donkey anti rabbit IgG (Life Technologies 21206).

For image quantification of neuronal cultures, methods were adapted from White et al. [19], green (β-III tubulin) and red (GFAP) channels were separately measured by setting a signal threshold and determining the area covered, representing neurite density for β-III-tubulin, and astrogliosis for GFAP. To quantify nuclear translocation of NF-kB, methods were adapted from Tatro et al. [21], the cells were delineated by creating a mask for the HLA-DR signal representing the cell bodies and the nuclei were delineated by creating a mask for the DAPI signal. The NF-kB signal was calculated for the cell body and the nuclei by total signal intensity, and then the percent-nuclear and percent-cytoplasm were calculated.

### Cytokine production assays

Cytokines and chemokines were measured in technical duplicate and biological triplicate using 25 μL aliquots following manufacturers instructions of a 7-plex pro-inflammatory cytokine quantitation kit (Mesoscale Discovery K15008B, Rockville, MD, USA), measured on a Sector Imager 2400 instrument, and concentration determined from a manufacturer-supplied standards (Mesoscale Discovery C0049-2). The following molecules were quantitated: IFN-γ, IL-1β, IL-10, IL-12 p70, IL-6, IL-8, and TNF-α.

### Statistical analysis

For GFAP and β-III tubulin comparisons, Student’s t-test was used to compare RAL vs Control of area covered. For NF-kB measurements, percent nuclear was arcsine-transformed (to account for upper and lower limits, 0-100%) and each condition compared by Student’s t test. For cytokine production, linear regression using Least Squares of concentration vs. time for each condition (Control, HIV, RAL, HIV + RAL) was calculated (fitted to Equation 1), including an interaction term. Equation 1: [Cytokine] = β_0_ + β_1_Time + β_2_Condition + β_3_Condition*Time, we tested for effects of time (rate of secretion), main effect of Condition, and a Condition × Time interaction. β_3_ corresponds to the effect that a given condition has on the cytokine secretion compared to the null hypothesis, P-values reported to test for a Condition × Time interaction is the probability that rate is the same for all conditions together. The rates of cytokine production (pg/mL⋅d) for each treatment condition were compared to Control by Dunnett’s test.

## Results and discussion

In this study, we measured cytokine secretion from human microglia under four conditions, 1) No-treatment control, 2) Infected with HIV, 3) Infected with HIV but treated with 20 nM RAL, and 4) 20 nM RAL alone. The concentration of 20 nM RAL was chosen on the basis of evaluating several pharmacokinetics studies. The mean trough plasma concentration from RAL once-daily parmacokinetics studies was 40 nM [22], while an independent study calculated a CSF:Blood Plasma ratio of 0.058 with a median (over 24 hr) plasma concentration 540.7 nM (31.36 nM in CSF) [23]. One additional study evaluated *intracellular* RAL concentration and calculated a 24 hr area under the curve (AUC) 1,884 nMxh [24], which is averages to roughly 78.5 nM. Therefore 20 nM RAL seemed to be a reasonably relevant concentration to assess in vitro effects on microglia.

**Figure 1:**
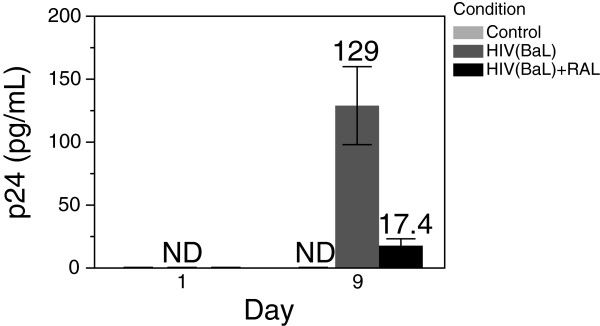
**HIV p24 measured in supernatant.** Microglia were exposed for 4 hr to stock HIV_BaL_ virus (equivalent to 5,000 pg/mL of p24), then washed in PBS. Aliquots of extracellular supernatant were then removed immediately (day 1 on plot) and after 9 d in culture, then p24 measured by ELISA. Plotted are means of three biological replicates with standard deviation. ND — not detected. P24 was was significantly higher in HIV + than HIV + RAL at 9 d (P < 0.001), and HIV + RAL was not significantly different from uninfected at 9 d (P = 0.19), by ANOVA and Tukey Honestly Significant Difference test.

**Figure 2:**
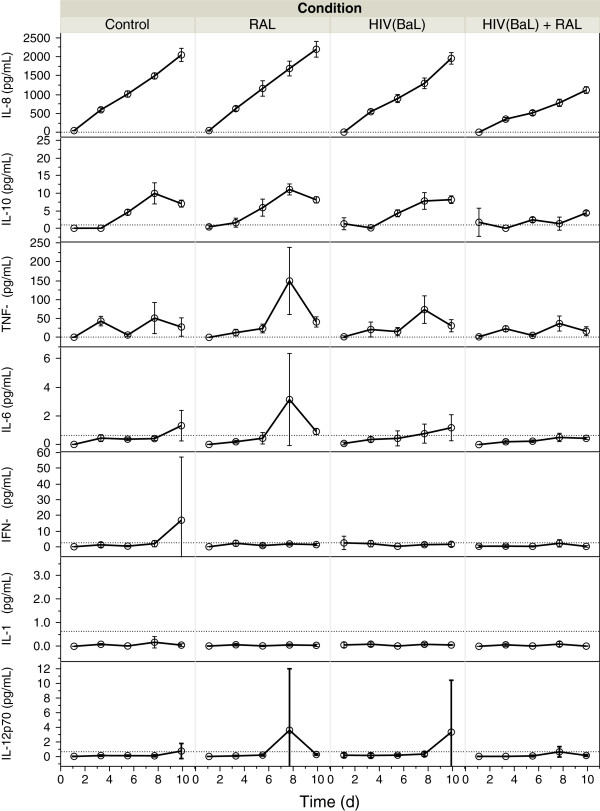
**IL-8, IL-10, TNF-α, IL-6, IFN-γ, IL-1β, and IL12p70 production in supernatant of microglia during 9 d in culture.** Microglia were infected with HIV or not, and exposed to 20 nM RAL immediately afterwards (RAL, HIV + RAL), and a non-treated culture from the same source grown alongside (Control). Supernatants were withdrawn and cytokines measured by Mesoscale Discovery 7-plex pro-inflammatory cytokine kit. Plotted are concentrations (pg/mL) vs. Time after infection, separated by treatment group, error bars indicate standard deviation of biological triplicates, dotted horizontal line indicates detection limits. Based on Standard Least Squares linear regression, RAL-treatment in HIV-infection significantly reduced the secretion rate of IL-8, IL-10, and TNF-α. In RAL-alone, there was higher TNF-α and IL-6 at day 8.

We found that RAL administered at 20 nM was effective at suppressing HIV infection in microglia (Figure 1) for at least 9 d. Only IL-8, IL-10, and TNF-α were above the detection limit. We calculated the rate of cytokine production for all three cytokines across the different treatment groups (Table 1). The mean IL-8, IL-10, and TNF-α concentrations and linear regression, separated by conditions are shown in Figure 2. For IL-10, IL-8 and TNF-α, there was a significant effect of RAL on the cytokine production in the context of HIV infection. Alone, the RAL-treated microglia had the highest concentration of IL-8, IL-10, and TNF-α. However, in the context of HIV infection, RAL-treated microglia had lowest production of TNF-α and IL-8. This makes sense because TNF-α autocrine signaling leads to IL-8 production via NF-kB. However, it is important to note that the highest production of TNF-α was in the presence of RAL alone (Table 1). IL-8 and IL-10 production were lowest in RAL treated microglia in the context of HIV, but not significantly different among the other groups (Table 1).

**Table 1:** **The rate of cytokine secretion +/- standard error over 9 days by microglia infected with HIV and / or treated with 20 nM RAL (pg × mL**^**-1**^ **× day**^**-1**^**)**

***Condition***	***TNF-α***	***IL-10***	***IL-8***
Control	3.09 ± 1.8	1.2 ± 0.16	245 ± 6.2
HIV	5.54 ± 1.8	1.07 ± 0.12	232 ± 8.1
RAL	10.9 ± 3.8	1.25 ± 0.15	268 ± 9.9
HIV + RAL	2.2 ± 1.0	0.33 ± 0.14	132 ± 5.0
^**a**^*P*-value	0.04	< 0.001	< 0.0001

^a^For a significant effect of treatment Condition on the rate of cytokine secretion against the null hypothesis that all treatments were the same.

It is remarkable that HIV and RAL alone induced cytokine expression, but in combination, was below control levels. This implies that RAL may be pro-inflammatory alone, and in the presence of HIV replication complex it is anti-inflammatory. One possibility is off-target effects on DNA-binding proteins, mimicking NF-kB - like activation, however in the presence of HIV-replication complex, it is bound and not interacting with off-target proteins.

We assessed cytotoxicity through measuring LDH-release in HIV-infected cells exposed to RAL and found background levels cytotoxicity due to HIV-infection, which was neither enhanced or diminished by RAL. The mean ± standard deviation cytotoxicity in HIV-alone was 43 ± 7.9%; and for HIV + RAL was 45 ± 3.2%. This may have been due to the protocol, which was acute infection via exposure to high levels of HIV for 4 hr, followed by media change including the drug. RAL may have suppressed further integration and infection of more cells with HIV, but it did not prevent lysis/death of those already infected.

Activation of the IL-8 gene is enhanced by signaling from NF-kB, and based on reduced IL-8 secretion in the HIV + RAL treated cultures, we hypothesized lower NF-kB activation and quantitated the nuclear translocation of NF-kB after 9 d infection, we calculated the percent-nuclear NF-kB compared to total NF-kB. The proportion of NF-kB localized to the nucleus was significantly lower in the HIV + RAL than the HIV + alone. Nuclear localization of phosopho-NF-kB was quantitated as percent-nuclear, then arcsine transformed for comparison by Dunnett’s test vs. Control. RAL alone (21%) and HIV + RAL (22%) were not significantly different from Control (20%), while HIV + alone was significantly higher from Control (28%, *P* = 0.02).

**Figure 3:**
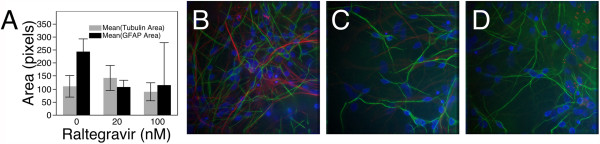
**Primary human neuron-glia cultures exposed to RAL. (A)** Quantification of area of β-III-tubulin (average area, μm^2^), and GFAP in neuron - glia cultures exposed to 0, 20, and 100 nM RAL for 15 hr. Representative images of cultures treated with **(B)** Control, **(C)** 20 nM, and **(D)** 100 nM RAL. Cultures were maintained for four weeks in neurobasal media on glass coverslips then exposed to RAL overnight, cells were fixed and stained for β-III tubulin (green) and GFAP (red) to illustrate neurons and astrocytes, respectively. Ten images were acquired by deconvolution fluorescence microscopy under 40X objective magnification and quantified using Image Pro Plus.

There are several cellular markers of neurons and astrocytes in cell culture, one of which is class-III β-tubulin, a structural microtubule protein specifically expressed in the cell body, axon, and dendrites of neuronal cells. To determine the neurotoxicity, we measured the area of coverage of β-III tubulin, similar to Robertson et al. [13], because retraction or loss of neurites would be detected by this method. In order to measure the relative amount of astrocytes in the culture, we similarly measured the area covered by the astrocyte-specific protein, GFAP. Diffuse astrocytosis was observed during HIV associated dementia and HIV-encephalitis [25], and in the CNS of transgenic mice expressing HIV proteins [26] and is a marker of astrogliosis or astrocytosis. In the absence of RAL, neuronal cultures had 110 ± 41 μm^2^ β-III tubulin and 244 ± 49 μm^2^ GFAP. While with 20 and 100 nM RAL, there was no significant effect on β-III tubulin, with 142 ± 48 and 89.4 ± 34 μm^2^, respectively, with P = 0.17 and P = 0.43 as assessed by Dunnett’s test vs. Control. However, with 20 and 100 nM RAL, there was a significant effect on GFAP, with 107 ± 26 and 115 ± 163 μm^2^, respectively, with P = 0.0094 and P = 0.01 as assessed by Dunnett’s test vs. Control. Thus, RAL had no significant effect on β-III tubulin area at 20 or 100 nM and an inhibitory effect on GFAP area. The possibility that RAL is toxic to astrocytes should be noted, considering the important role that they play in maintaining the blood brain barrier and neuronal maintenance. Figure 3 shows the quantitation of β-III tubulin and GFAP area as well as representative photomicrographs.

The CNS has been proposed as a compartment for a latent HIV reservoir which has long-term neurological effects with downstream neurocognitive consequences, attributed to cytokine production by infected perivascular CNS macrophages and parenchymal microglia [10]. Therefore, understanding whether antiretroviral compounds themselves are neurotoxic becomes important when infected individuals are on therapy for decades. The purpose of the present study was to assess whether RAL, an inhibitor of strand-transfer, has neurotoxic properties and whether RAL slows the rate of cytokine secretion by HIV infected microglia. We found that RAL is not neurotoxic at 20 or 100 nM and that it significantly inhibits cytokine secretion in HIV infected microglia in vitro.

Robertson *et al.*[13] found that exposure of primary human neurons caused statistically significant reduction of MAP2, a dendritic marker of mature neurons, when exposed to some antiretroviral drugs. Individually, abacavir, 2′,3′-dideoxyinosine, and nevirapine are predicted to have relatively high risk of neurotoxicity, with at least a 10% drop in MAP2 intensity at observed typical cerebrospinal fluid (CSF) concentrations of the drugs [13]. Median RAL concentration in the CSF of treated HIV patients was observed to be 14.5 ng/mL (30.05 nM) [23], which is within range of the concentrations we tested for neurotoxicity. This suggests that RAL has relatively low neurotoxic risk.

We measured cytokine production by withdrawal of supernatant from infected microglia cultures and quantification using a multiplex assay and found production of IL-8, IL-10, and TNF-α, which increased linearly. RAL on its own caused significant increase in the rate of production of these three cytokines, mainly IL-10, an anti-inflammatory cytokine; but resulted in a significant decrease when administered with HIV. The most abundant was IL-8, a molecule with potent chemotaxic properties for neutrophils [27] and monocytes [28]. IL-8 gene transcription is induced by NF-kB activation and nuclear translocation, and is dependent on TNF-α. One preliminary positron emission tomography (PET) study found slightly increased retention of the PET ligand ^11^C-PK11195, which binds to activated microglia, in neurologically asymptomatic and ARV-treated HIV-infected individuals [11]. One human study assessed IL-8 concentration in CSF of patients with HIV associated dementia compared to HIV-infected patients with no neurocognitive impairment and found higher IL-8 in CSF of those with dementia [29]. In our experiments, phospho-NF-kB nuclear translocation was significantly higher only in the HIV-infected cultures, not in Control, RAL-alone, or HIV + RAL.

We also observed production of IL-10, an anti-inflammatory cytokine which inhibits T cell proliferation and is putatively produced early in HIV infection via NF-kB signaling [30], which would be an antiviral action, allowing the incipient reservoir to avoid detection.

In comparison with nucleoside reverse transcriptase inhibitors, tenofovir and zidovudine, RAL has relatively modest effects on monocyte cytokine production. Zidovudine dose-dependently increased secretion of IL-8 and tenofovir decreased it. Likewise, the anti-inflammatory IL-10 was reduced in the presence of tenofovir while zidovudine did not affect it [31].

One possible explanation for reduced TNF-α, IL-8, and IL-10 production in our cultures is simply due to fewer infected cells. Another possible explanation is that inhibition of the strand transfer step of HIV integration prevents the DNA damage and repair process from being initiated, which may lead to downstream microglial activation. One weakness to the present study is the lack of comparison with another antiretroviral compound or lack of testing for combinations. Additionally, with measuring the cytokines at one-day intervals, it is difficult to determine the order of events except by interpolating from what is already known. An important future experiment would be to assess the effect of conditioned media from brain macrophage cultures on neuronal and glia cultures.

## Conclusions

Results from this study suggest a low probability for neurotoxicity of RAL and likely neuroprotective effect in HIV-infection by suppressing the production of chemotaxic inflammatory cytokine, IL-8.

## Abbreviations

HIV human immunodeficiency virus: RAL, Raltegravir; IL: Interleukin; IFN: Interferon; TNF: Tumor necrosis factor; NF: Nuclear factor; HLA-DR: Human leukocyte antigen DR; GFAP: Glial fibrillary acidic protein; CSF: Cerebrospinal fluid.

## Competing interests

ETT, BS, and CLA received support from a grant to University of California San Diego by Merck & Company. SL declares no conflicts of interest.

## Authors’ contributions

CLA participated in study design, conception; SL participated in study design and conception, and manuscript editing; BS performed experiments, analysis, and microscopy; ETT performed data analysis, experiments, and wrote the manuscript. All authors read and approved the final manuscript.

## Pre-publication history

The pre-publication history for this paper can be accessed here:

http://www.biomedcentral.com/1471-2334/14/386/prepub
